# Topology and Sequence-Dependent Micellization and Phase Separation of Pluronic L35, L64, 10R5, and 17R4: Effects of Cyclization and the Chain Ends

**DOI:** 10.3390/polym14091823

**Published:** 2022-04-29

**Authors:** Tomohisa Watanabe, Yubo Wang, Tomoko Ono, Satoru Chimura, Takuya Isono, Kenji Tajima, Toshifumi Satoh, Shin-ichiro Sato, Daichi Ida, Takuya Yamamoto

**Affiliations:** 1 Graduate School of Chemical Sciences and Engineering, Hokkaido University, Sapporo 060-8628, Hokkaido, Japan; tomohisa_watanabe@eis.hokudai.ac.jp (T.W.); ougyokuhaku@eis.hokudai.ac.jp (Y.W.); a1fi_7ht1@outlook.jp (S.C.); 2 Division of Applied Chemistry, Faculty of Engineering, Hokkaido University, Sapporo 060-8628, Hokkaido, Japan; t-ono1808@eng.hokudai.ac.jp (T.O.); isono.t@eng.hokudai.ac.jp (T.I.); ktajima@eng.hokudai.ac.jp (K.T.); satoh@eng.hokudai.ac.jp (T.S.); s-sato@eng.hokudai.ac.jp (S.S.); 3 Department of Polymer Chemistry, Graduate School of Engineering, Kyoto University, Katsura 615-8510, Kyoto, Japan; ida@molsci.polym.kyoto-u.ac.jp

**Keywords:** cyclic polymer, block copolymer, Pluronic, micellization, cloud point, phase transition

## Abstract

The topology effects of cyclization on thermal phase transition behaviors were investigated for a series of amphiphilic Pluronic copolymers of both hydrophilic–hydrophobic–hydrophilic and hydrophobic–hydrophilic–hydrophobic block sequences. The dye solubilization measurements revealed the lowered critical micelle temperatures (*T*_CMT_) along with the decreased micellization enthalpy (Δ*H*_mic_) and entropy (Δ*S*_mic_) for the cyclized species. Furthermore, the transmittance and dynamic light scattering (DLS) measurements indicated a block sequence-dependent effect on the clouding phenomena, where a profound decrease in cloud point (*T*_c_) was only found for the copolymers with a hydrophilic–hydrophobic–hydrophilic block sequence. Thus, the effect of cyclization on these critical temperatures was manifested differently depending on its block sequence. Finally, a comparison of the linear hydroxy-terminated, methoxy-terminated, and cyclized species indicated the effect of cyclization to be unique from a simple elimination of the terminal hydrophilic moieties.

## 1. Introduction

The investigation into the properties of polymers with non-linear architecture, namely, topological polymers, has revealed various unique structure–property relationships [1]. Amongst them, polymers having a cyclic topology have especially been the subject of attention from both synthetic and physical property viewpoints, resulting in extensive reviews, highlights [2,3,4,5,6,7,8,9], and more recently, application-oriented researches [10,11,12,13,14]. Naturally, the effects of the cyclic topology have been investigated beyond simple homopolymers to self-assembling block copolymer systems, where the thermodynamics and structural properties of self-assembly phenomena were found to be affected by the geometrical constraints induced from the cyclic topology [15,16,17,18]. In general, cyclic copolymer amphiphiles were found to assemble micelles having reduced dimensions and higher densities compared to their corresponding linear counterparts [16,17,19]. In other examples, the morphology of the assembled micelles differed depending on the topology of the polymers [20,21]. Moreover, enhancement in thermal and salt stability was found for cyclized copolymer micelles compared to their corresponding linear species, resulting from the inhibition of agglomeration induced by chain-bridging of micelles [22].

The effects of polymer topology are, in theory, not bound by its chemical structure. However, physical properties exhibited by actual polymer systems are affected by a variety of factors of physical and/or chemical origin, thus complicating the elucidation of the topology effects. For this reason, polymers with a wide understanding of the relations between physical properties and structural parameters such as molecular weight, block ratio, and block sequence are attractive candidates for an investigation into the effects of polymer topology. In this context, symmetrical triblock copolymers comprised of hydrophilic poly(ethylene glycol) (PEG) and hydrophobic poly(propylene glycol) (PPG) blocks, commercially registered under trademark names of Pluronic [23] are ideal due to their well-studied nature [24,25]. Pluronic copolymers can be classified into two types based on their block sequence. Copolymers with the PEG–PPG–PEG sequence are widely known as ABA-type Pluronic, with the hydrophobic PPG block sandwiched between two hydrophilic PEG blocks. In contrast, copolymers with a PPG–PEG–PPG sequence are known as “reverse” or BAB-type Pluronic, consisting of a middle hydrophilic PEG block with two terminal hydrophobic PPG blocks. Attention has been focused on the property–structure relationships regarding parameters, such as molecular weight and block ratios [26,27,28,29,30], where, for example, micellization conditions were found to be more significantly influenced by the length of the PPG blocks compared to the PEG blocks [30]. While fewer studies have focused on the block sequence, direct comparisons between ABA-type and BAB-type Pluronic copolymer solutions revealed reduced self-assembling tendencies for the latter, owing to the looped geometry of the copolymers required upon micellization [31,32].

To date, only a single investigation conducted by Booth and coworkers has focused on the physical properties of cyclized Pluronic copolymers [33], apart from our recent report [34]. In their work, cyclization was achieved for a single relatively large Pluronic, EG_52_–PG_34_–EG_52_, via acetalization. The cyclization for this Pluronic species, however, did not confer a significant effect upon its micellization conditions or micelle hydrodynamic radii, while a more notable increase in the aggregation number (*N*_agg_) was found. Our more recent study reported the synthesis and interfacial properties of cyclized Pluronic copolymers of diverse chemical compositions, revealing an enhancement in interfacial activity via cyclization to be a prevalent phenomenon [34]. Furthermore, the degree of enhancement in interfacial activity upon cyclization was found to be influenced by the block ratio and sequence of the linear species, with a prominent difference in the linear and cyclic species arising in surfactants having a large PEG block composition. Since our previous study focused on the effect of cyclization at the air–water interface, its micellization and phase transition properties were not investigated. However, the obtained results suggest the cyclic topology affects the interfacial properties of the solutions, albeit with different magnitudes depending on their structure and composition. It is, therefore, of importance to expand the work and conduct a comprehensive study into the bulk solution properties of various cyclized Pluronic copolymers in order to elucidate the effects of the cyclic topology in relation to other chemical parameters.

This work presents a comprehensive investigation into the effects of cyclization on the temperature-induced aggregation phenomena of various Pluronic copolymers. The determination of critical micellization temperatures (*T*_CMT_) through dye solubilization and cloud point (*T*_c_) through light transmittance revealed distinct effects of cyclization. For example, cyclized species were found to display generally lower *T*_CMT_ compared to their linear counterparts. Furthermore, thermodynamic studies revealed reduced micellization enthalpy (Δ*H*_mic_) and entropy (Δ*S*_mic_) for the cyclized species. Both *T*_C__MT_ and the thermodynamic parameters of micellization were more heavily affected upon cyclization for the copolymers with a BAB-type block sequence. The *T*_c_, on the other hand, was more significantly affected upon cyclization for the ABA-type copolymers, resulting in prominently reduced values. The obtained results suggest that cyclization influences various phase stability of Pluronic copolymers in relation to their block sequence.

## 2. Materials and Methods

### 2.1. Materials

CH_3_I (>99.5%), KOH (>85.5%), NaCl (>99.0%), Na_2_SO_4_ (>99.0%), CH_2_Cl_2_ (>99.0%), MeOH (>99.5%), and CHCl_3_ (>99.0%) were purchased from Kanto Chemical Co., Inc.,Tokyo, Japan, while TsCl (>98.0%) and chlorobenzene (>99.0%) were purchased from Junsei Chemical Co., Ltd.,Tokyo, Japan, and Nacalai Tesque, Inc., Kyoto, Japan, respectively. All reagents of the above-mentioned were used as received. *n*-Hexane (>99.0%) was purchased from Kanto Chemical Co., Inc., Tokyo, Japan, and purified by distillation before use. Dehydrated tetrahydrofuran (THF), stabilizer free (>99.5%), and dehydrated dichloromethane (CH_2_Cl_2_) (>99.5%) for the reactions were purchased from Kanto Chemical Co., Inc., Tokyo, Japan, and purified using a solvent purification system (MB-SPS-Compact, MBRAUN, Garching, Germany). Poly(ethylene glycol)–*block*–poly(propylene glycol)–*block*–poly(ethylene glycol) (Pluronic L35, *M*_n_~1900), poly(ethylene glycol)–*block*–poly(propylene glycol)–*block*–poly(ethylene glycol) (Pluronic L64, *M*_n_~2900), poly(propylene glycol)–*block*–poly(ethylene glycol)–*block*–poly(propylene glycol) (Pluronic 10R5, *M*_n_~1700), poly(propylene glycol)–*block*–poly(ethylene glycol)–*block*–poly(propylene glycol) (Pluronic 17R4, *M*_n_~2700) were purchased from Sigma–Aldrich (Merck KGaA, Darmstadt, Germany) and poly(ethylene glycol) (PEG), (*M*_n_~2000) was obtained from Tokyo Chemical Industry, Co., Inc., Tokyo, Japan. The linear polymers were purified using preparative SEC. Pluronic L35 and L64 were further purified by a previously reported method [35,36]. Thus, 1.0 g of the copolymers was vigorously stirred in 100 mL of *n*-hexane at room temperature for 15 min, and the supernatant *n*-hexane phase was removed. While the majority of the copolymer remained undissolved, hydrophobic polymeric impurities were extracted into the *n*-hexane phase and thus removed through this procedure. This purification procedure was repeated three times, and the copolymer was dried under reduced pressure overnight.

### 2.2. Nuclear Magnetic Resonance Spectroscopy

A JNM-ESC400 instrument (JEOL Ltd., Tokyo, Japan) was used to measure proton (^1^H, 400 MHz) and carbon (^13^C, 100 MHz) nuclear magnetic resonance (NMR) spectra using CDCl_3_ at room temperature.

### 2.3. Size Exclusion Chromatography

Size exclusion chromatography (SEC) measurement was carried out using a PU-980 Plus pump (JASCO Co., Tokyo, Japan) equipped with KF-804L columns (8.0 mm × 300 mm × 2, Shodex) and a KF-G guard column (Shodex, Tokyo, Japan) inside a CO-2065 Plus column oven (JASCO Co., Tokyo, Japan) set at a temperature of 40 °C. An RI-2031 Plus differential refractometer (JASCO Co., Tokyo, Japan) was used as a detector, and THF as an eluent was set at a flow rate of 1.0 mL/min.

### 2.4. Preparative SEC

Fractionation using preparative SEC was carried out using a Japan Analytical Industry LC-908 recycling preparative HPLC equipped with a RI detector RI-5 (JAI. Co., Ltd., Tokyo, Japan) and L-7110 pump (Hitachi, Ltd., Tokyo, Japan) and JAIGEL columns (2H, 3H, and a pre-column, JAI. Co., Ltd., Tokyo, Japan) connected in series. Filtered CHCl_3_ as eluent was set at a flow rate of 3.5 mL/min.

### 2.5. Synthesis of Cyclized Polymers

The intramolecular cyclization of PEG homopolymer and Pluronic copolymers having hydroxy end groups was performed in accordance with previous reports [33,34,37]. For PEG and ABA-type copolymers, cyclization was carried out by the Williamson-ether synthesis. Typically, the reaction was conducted by gradually adding 50 mL of a THF solution of Pluronic L35 (5.0 g, 1 equiv. mol) and TsCl (1.3 equiv. mol) at a rate of 20 μL/min into a THF/*n*-hexane (100 mL, 70/30 *v*/*v*) suspension of KOH (5.0 g) at 40 °C under an Ar gas atmosphere. The mixture was additionally stirred for 2 d at the same temperature. After filtration, the reaction mixture was concentrated under reduced pressure overnight. The obtained residue was dissolved in CH_2_Cl_2_ and washed thrice using saturated NaCl aqueous solution. Residual water in the organic phase was removed using Na_2_SO_4_ and was further dried overnight under reduced pressure. Silica gel column chromatography of the residue was carried out using a CHCl_3_/MeOH (90/10 *v*/*v*) mixture, to give 2.4 g of a crude product. Finally, 900 mg of the crude product was injected in a preparative recycling SEC to fractionate cyclized species from intermolecularly reacted byproducts and the linear precursors, to give 360 mg of pure cyclized Pluronic L35 (***c*-L35**). Meanwhile, cyclization for BAB-type Pluronic copolymers was carried out through the acetalization reaction. Thus, typically, 5.0 g of Pluronic 10R5 was dissolved in 50 mL of CH_2_Cl_2_, and the solution was added into a KOH (5.0 g) dispersion of a CH_2_Cl_2_/*n*-hexane mixture (100 mL, 65/35 *v*/*v*) at a rate of 12.5 μL/min. The mixture was stirred at 40 °C for an additional 3 d. After filtration, the reaction mixture was concentrated under reduced pressure. Redissolution in CH_2_Cl_2_, three times washing of the residue using brine, and silica gel column chromatography (eluent; CHCl_3_/MeOH, 90/10 *v*/*v*) was used to isolate 1.7 g of a crude product. Finally, 500 mg of the crude product was injected into a preparative recycling SEC to give 170 mg of pure cyclized Pluronic 10R5 (***c*-10R5**).

### 2.6. Synthesis of Chain-End Methylated Polymers

Methylation of chain-ends for ABA and BAB-type Pluronic copolymers was carried out following previous reports [10,34]. Typically, 50 mL of a chlorobenzene solution of Pluronic L35 (2.5 g, 1.0 equiv.) was added (70 μL/min) to a 100 mL chlorobenzene suspension of KOH (5.0 g) and CH_3_I (0.56 g, 3.0 equiv.) under an Ar gas atmosphere at room temperature. The mixture was stirred additionally for 24 h and purified through filtration and silica gel column chromatography in CHCl_3_/MeOH (90/10 *v*/*v*) to give 1.9 g of methoxy-terminated Pluronic L35 (***l*-L35(OMe)**).

### 2.7. Preparation of Pluronic Copolymer Solutions

Required amounts of copolymer were dissolved in Milli-Q water and stirred at room temperature for over 12 h. The samples were kept in a refrigerator in tightly closed glass vials for over 48 h for complete dissolution and filtered through a 0.45 μM Millipore filter prior to measurement.

### 2.8. Critical Micelle Temperature (T_CMT_) by Dye Solubilization

A dye solubilization technique using 1,6-diphenyl-1,3,5-hexatriene (DPH) was used to determine *T*_CMT_ for the Pluronic copolymer solutions [30]. First, copolymers were dissolved in Milli-Q water and stirred at room temperature for over 12 h. 25 μL of a 0.4 mM DPH/methanol solution was added into 2.5 mL of the copolymer aqueous solution. The final sample solution contained 1 vol% of methanol and 4.0 μM DPH, at four copolymer concentrations (*c*) of 0.30, 1.0, 3.0, and 10 g/L. The sample solutions were kept in the dark for at least 3 h prior to measurement, to ensure complete mixing equilibrium of the system. Each sample solution was heated at a rate of 0.1 °C min^−1^, and absorption spectrum (340–400 nm) was measured in 2 °C increments after an equilibration time of 10 min at each temperature. The absorption intensity changes upon temperature elevation at the maximum absorption wavelength of DPH (*λ*_max, DPH_ = 356 nm) were used in the determination of its *T*_CMT_.

### 2.9. Cloud Point (T_c_) Measurement

Transmission of the solution (%T) was measured at 600 nm on a V-670 UV–Visible spectrophotometer (JASCO Co., Tokyo, Japan) using an M25-UV-2 micro quartz cell (GL Science Inc., Tokyo, Japan). Aqueous solutions of the Pluronic copolymers were stirred at 60 rpm inside the spectrophotometer and heated at a rate of 1 °C/min. %T was measured in 1 °C increments. The lowest temperature at which %T became 90% or less was determined as *T*_c_. For the PEG samples, aqueous solutions of NaH_2_PO_4_ at a concentration of 250 g/L were used for dissolution of the polymer to induce phase separation below the boiling point of water [38,39].

### 2.10. Dynamic Light Scattering (DLS)

DLS measurements were performed on a Malvern Zetasizer Nano instrument equipped with a 50 mW frequency-doubled DPSS Nd:YAG laser (*λ* = 532 nm) (Malvern Panalytical, Ltd., Malvern, UK). The light scattering signal was obtained at a fixed angle of 173°. Aqueous solutions of Pluronic L64 and its derivatives (10 g/L) were measured at various temperatures after an equilibration time of 10 min. Non-negative least squares analyses [40,41] provided in software built into the instrument were used to determine the number distribution of the apparent hydrodynamic diameter at finite concentration.

## 3. Results and Discussions

### 3.1. Preparation of Sample Polymers and Their Solutions

The cyclized PEG homopolymer and the Pluronic copolymers L35, L64, 10R5, and 17R4 were synthesized and purified according to a reported method [33,34,37], through intramolecular cyclization of the corresponding linear prepolymers. In the case of Pluronic L35, the starting prepolymer with hydroxy chain ends and its products are named ***l*-L35(OH)** and ***c*-L35**, respectively, in this paper. The other polymers are expressed accordingly. For the PEG homopolymers and PEG–PPG–PEG (ABA-type) Pluronic L35 and L64, the Williamson-ether synthesis was undertaken for the reaction between the chain-end hydroxy groups. For PPG–PEG–PPG (BAB-type) Pluronic 10R5 and 17R4, on the other hand, an acetalization reaction was carried out instead, due to reduced reactivity of the secondary alcohol at the chain ends. Linear polymers with methylated chain ends were also prepared via a reaction with iodomethane in order to clarify the extent of the effect of the cyclic topology from chemically induced changes due to the elimination of the chain-end hydroxy groups. In the case of L35, the methylated copolymer is named ***l*-L35(OMe)**, and the others are named accordingly. The successful cyclization and dimethylation were confirmed through size-exclusion chromatography (SEC) (Figure S1) and NMR (Figures S2 and S3). The detailed characterization of the synthesized polymers has been reported elsewhere [34].

In the case of L64, the commercial product (linear prepolymer) is known to contain a certain number of polymeric impurities of a stronger hydrophobic nature. These polymeric impurities are known to substantially affect their solution properties at conditions close to micellization, with a number of studies reporting their removal [35,36]. Accordingly, the repeated washing of the prepolymer using *n*-hexane successfully purified the prepolymer, as confirmed through the temperature-dependent transmission of the polymer solution (Figure S4). A spike in %T that was observed at around 40 °C before purification due to the aggregation of the impurities clearly disappeared for a solution prepared from purified ***l*-L64(OH)**. The cyclization and methylation reactions were both carried out using the purified copolymer. The same procedure was carried out for the purification of ***l*-L35(OH)**.

**Figure 1:**
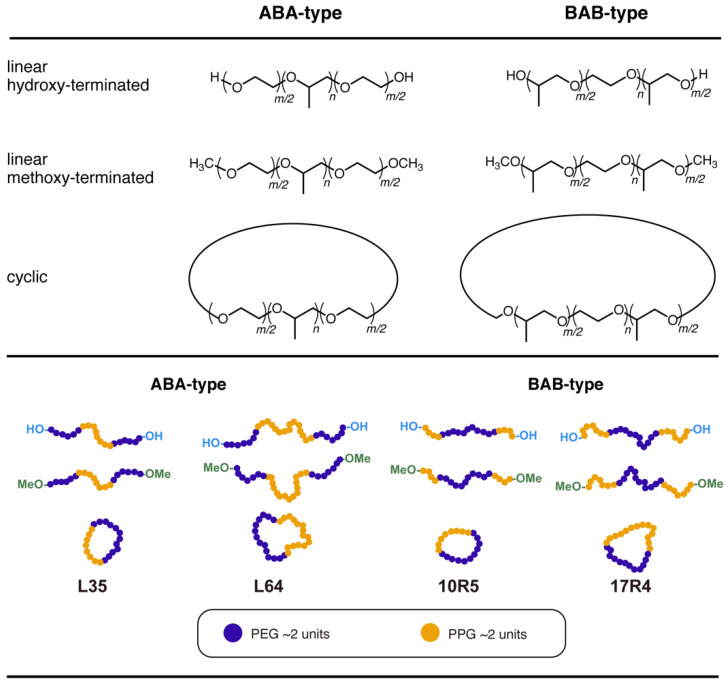
Chemical structures (**top**) and schematic illustrations (**bottom**) of various Pluronic copolymers used in this study. Both ABA- and BAB-type copolymers were compared to clarify the effect of cyclization in relation to their block sequence.

Among the various types of Pluronic copolymers commercially available, L35, L64, 10R5, and 17R4 were selected since their total molecular weight and block composition cause aggregation at relatively mild conditions (Figure 1). In addition, a copious amount of research has been conducted for ABA-type Pluronic copolymers, especially L64, revealing detailed aspects of their aggregation behavior [35,42,43,44,45,46]. Reverse-type or BAB-type Pluronic copolymers, on the other hand, have received less attention, with fewer reports on their physical properties [47,48]. However, the block sequential distinction between the hydrophilic–hydrophobic–hydrophilic ABA-type and the hydrophobic–hydrophilic–hydrophobic BAB-type results in surprisingly contrasting properties, such as substantially higher critical micellization concentrations for the latter [32], even for copolymers with a similar molecular weight and hydrophilic/hydrophobic block composition. Therefore, a comparison of the changes in solution properties between ABA-type and BAB-type Pluronic copolymers allows a clarified understanding of the effect of cyclization in relation to the block sequence.

The aqueous solutions of the linear polymers with either hydroxy chain ends (***l*-L35(OH)**, ***l*-L64(OH)**, ***l*-10R5(OH)**, ***l*-17R4(OH),** and ***l*-PEG(OH)**) or methoxy chain ends (***l*-L35(OMe)**, ***l*-L64(OMe)**, ***l*-10R5(OMe),** and ***l*-17R4(OMe)**) along with their cyclized products (***c*-L35**, ***c*-L64**, ***c*-10R5**, ***c*-17R4,** and ***c*-PEG**) were prepared by dissolving an appropriate amount of the copolymers in Milli-Q water. The copolymer solutions were stirred for over 12 h at room temperature and kept in a refrigerator for over 48 h for complete dissolution. Each solution was filtered immediately prior to measurement to remove any macroscopic impurities. For *T*_c_ measurements of the PEG homopolymers, a salting-out effect was utilized to induce phase separation below the boiling point of water [38,39]. Thus, ***l*-PEG(OH)** and ***c*-PEG** were dissolved in an aqueous solution of NaH_2_PO_4_ at a concentration of 250 g/L.

### 3.2. Critical Micellization Temperature (T_CMT_) by Dye Solubilization

**Figure 2:**
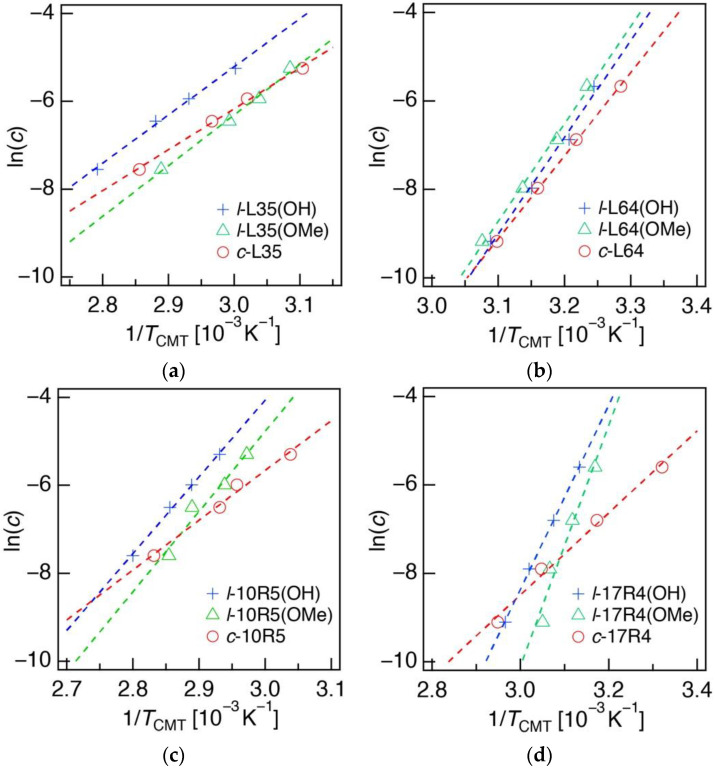
(**a**) ln(*c*) vs. 1/*T*_CMT_ plot for aqueous solutions of linear hydroxy-terminated (blue), methoxy-terminated (green), and cyclized (red) L35, indicated as ***l*-L35(OH)**, ***l*-L35(OMe),** and ***c*-L35**, respectively. Those of (**b**) L64, (**c**) 10R5, and (**d**) 17R4 are also shown. Enthalpy of micellization (Δ*H*_mic_) was calculated from the slope of the linear fitting of the plots.

In order to evaluate the thermal response behavior of the synthesized cyclic Pluronic copolymers, a *T*_CMT_ measurement through the hydrophobic dye solubilization technique, using 1,6-diphenyl-1,3,5-hexatriene (DPH) was performed [30]. *T*_CMT_ was obtained from the intersection temperature value at which a change in the slope of the absorption intensity of DPH (*λ*_max, DPH_ = 356 nm) was observed, indicating micelle formation and dye solubilization within the hydrophobic core (Figure S5). Furthermore, the enthalpy of micellization (Δ*H*_mic_) was calculated from the slope of the linear fitting of the ln(*c*) versus 1/*T*_CMT_ plots obtained at four polymer molar concentrations (*c*), in accordance with the following Equation (1) (Figure 2). Using the Δ*H*_mic_ values, Δ*G*_mic_ at *c* = 10 g/L and at *T*_CMT_, as well as Δ*S*_mic_, were calculated by the following Equations (2) and (3) [30]:

Δ*H*_mic_ = *R*[∂ln (*c*)/∂(1/*T*_CMT_)] (1)

Δ*G*_mic_ = *RT*_CMT_ ln (*X*) (2)

Δ*S*_mic_ = (Δ*H*_mic_ − Δ*G*_mic_)/*T*_CMT_ (3)

where *R* is the gas constant, and *X* is the copolymer concentration in mole fraction at the micellization condition.

For 10 g/L ABA-type copolymer solutions, *T*_CMT_ of ***l*-L35(OH)** was 58 °C, and that of ***c*-L35** decreased to 45 °C. The same trend was observed for L64, where cyclized species displayed comparably lower *T*_CMT_ than their corresponding linear counterpart with hydroxy chain-end groups (***l*-L64(OH)**, 35 °C; ***c*-L64**, 31 °C in Table 1). The methylation of the chain ends also resulted in lowered *T*_CMT_ for L35, but no significant effect was observed for L64 (***l*-L35(OMe)**, 51 °C; ***l*-L64(OMe)**, 36 °C in Table 1). The difference in the critical micellization conditions between cyclized and linear ABA-type Pluronic was rather pronounced in comparison to the report by Booth and coworkers, where they were unable to define a clear effect of cyclization on their critical micellization concentrations for both PEG–PPG–PEG and PEG–poly(butylene glycol)–PEG triblock copolymers [33,49]. A similar decrease in *T*_CMT_ was observed for the BAB-type copolymers; from 69 to 62 °C for 10R5 and from 46 to 28 °C for 17R4 upon cyclization. *T*_CMT_ of 10R5 and 17R4 also decreased upon methylation of the chain ends (***l*-10R5(OMe)**, 65 °C; ***l*-17R4(OMe)**, 42 °C in Table 1), suggesting the elimination of the terminal hydroxy groups of PPG to also lower their *T*_CMT_. In any case, the cyclized copolymers displayed the lowest *T*_CMT_, indicating the effect of topology to be more significant compared to the consequence of the simple elimination of hydrophilic terminal moieties.

When Δ*H*_mic_ was determined from the slope of the ln(*c*) versus 1/*T*_CMT_ plots, the smallest values were found for the cyclic species (Figure 2 and Table 1), suggesting decreased enthalpic inhibition against micellization. According to previous reports, smaller Δ*H*_mic_ values for cyclized ABA-type copolymers arise from the reduced exposure of the hydrophobic B segment to water in the unimer state [33,49,50]. Thus, the hydrophilic A segment in the cyclized form likely more effectively shielded the hydrophobic B segment from contact with water. Furthermore, when the ABA- and BAB-type copolymers are compared, a more significant effect on Δ*H*_mic_ was observed for the latter. For example, Δ*H*_mic_ of ***c*-10R5** and ***c*-17R4** were found to be drastically reduced to 94 and 77 kJ/mol, respectively, from that of their linear hydroxy-terminated counterparts (***l*-10R5(OH)**, 145 kJ/mol; ***l*-17R4(OH)**, 173 kJ mol^−^^1^). This was likely caused by the hydrophobic chain length to double upon cyclization for the BAB-type copolymers, where the Δ*H*_mic_ value per hydrophobic repeating unit is known to be smaller as the segment becomes longer due to the formation of tight coils, minimizing contact with water [51].

Similarly, smaller values for the entropy of micellization (Δ*S*_mic_) were found for the cyclized species. This can also be attributed to the effect of the cyclic topology on the conformations in the unimer state and its relation to the hydrophobic effect. The hydrophobic effect is an entropic driving force towards micellization, arising from the release of water molecules from the lowered entropic states due to contact with the hydrophobic segments of the amphiphiles in the unimeric state [52]. This entropically driven process is known to be responsible for the micellization of many amphiphilic molecules, including Pluronic copolymers [32], and the positive Δ*H*_mic_ and Δ*S*_mic_ values obtained for the copolymers in this work also indicate micellization of the linear and cyclized Pluronic to be entropically driven. However, as mentioned above, cyclization of the copolymers leading to an efficient shielding of the hydrophobic PPG blocks from the surrounding water environment is also expected to reduce the hydrophobic effect, thus resulting in a lower Δ*S*_mic_. In addition, the presence of “dangling chains” in the less structured micelles of the BAB-type Pluronic copolymers may contribute to the more drastic decrease in Δ*S*_mic_ upon cyclization (e.g., ***l*-10R5(OH)**, 505 J/mol K; ***c*-10R5**, 365 J/mol K) compared to the ABA-type copolymers (e.g., ***l*-L35(OH)**, 352 J/mol K; ***c*-L35**, 319 J/mol K) [50]. Monte Carlo simulations of the micellization of linear ABA- and BAB-type copolymers performed by Kim and Jo revealed the latter copolymers to possess larger Δ*S*_mic_ due to their less structured micelles and dangling chains [53,54]. Therefore, since cyclization of both ABA- and BAB-type copolymers result in an AB-type diblock copolymer, the Δ*S*_mic_ differences can rationally be expected to be more prominent for the copolymers with the BAB-type block sequence.

Interestingly, while the ABA-type Pluronic copolymers displayed comparable Δ*H*_mic_ and Δ*S*_mic_ values for the linear methoxy-terminated species to that of the linear hydroxy-terminated species, an evident increase in both the thermodynamic parameter values were found for 17R4, a copolymer with a BAB-type sequence and relatively long PPG blocks. This was indicative of a more significant influence of the hydroxy groups for sufficiently long hydrophobic PPG segments on the micellization phenomena compared to the hydroxy groups of the hydrophilic PEG segments or short PPG segments. The larger Δ*S*_mic_ values for the methylated species are hypothesized to result from the stronger hydrophobicity, and the larger Δ*H*_mic_ values may possibly result from relatively reduced hydrogen bonding interaction within the micelle core. Thus, the fraction of water known to be contained within the Pluronic micelle core [55,56] may have decreased by methylation of the chain-ends due to their stronger hydrophobic nature, resulting in a larger number of hydrogen bonding severances and a larger enthalpic change upon micelle formation.

### 3.3. Cloud Point (T_c_)

**Figure 3:**
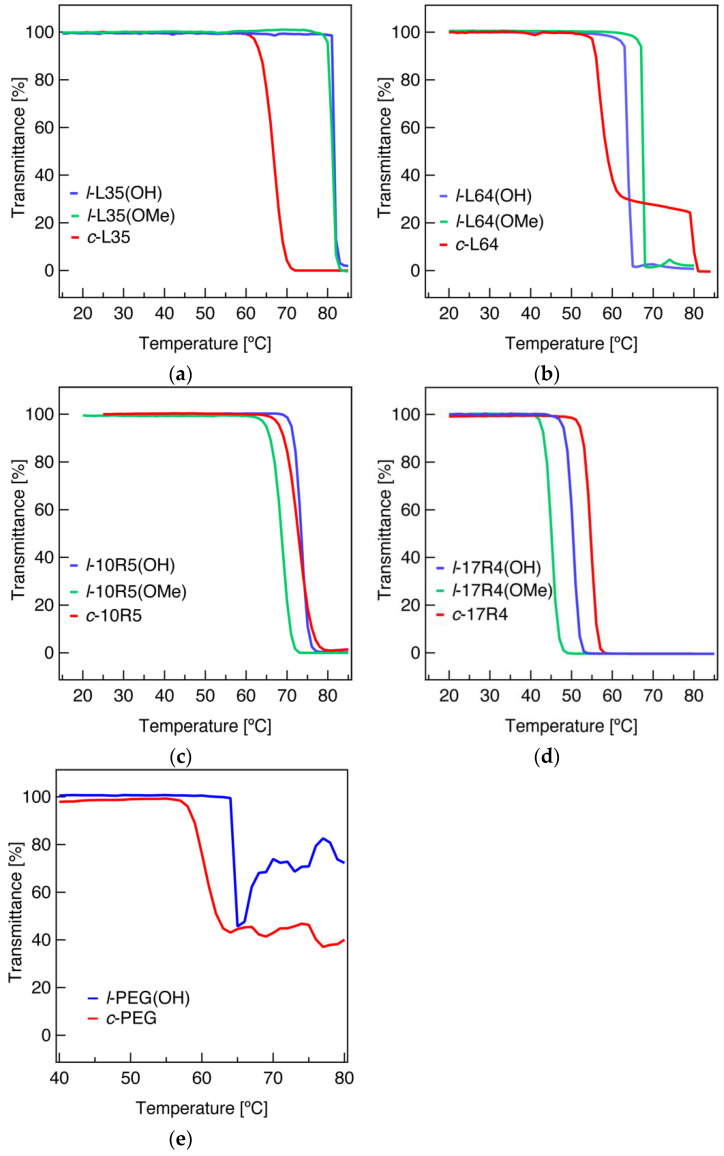
(**a**) Temperature-dependent transmittance (%T) at 600 nm for 10 g/L aqueous solutions of linear hydroxy-terminated (blue), methoxy-terminated (green), and cyclized (red) L35, indicated as ***l*-L35(OH)**, ***l*-L35(OMe),** and ***c*-L35**, respectively. Those of (**b**) L64, (**c**) 10R5 and (**d**) 17R4 are also shown. (**e**) %T at 600 nm for 10 g/L linear hydroxy-terminated (blue) and cyclized (red) PEG homopolymers in aqueous NaH_2_PO_4_ solutions at the concentration of 250 g/L.

The temperature-dependent transparency (%T) of Pluronic copolymer solutions at a concentration of 10 g/L was measured to determine the aggregation and phase separation behavior. Here, *T*_c_ was defined by the lowest temperature at which %T at the wavelength of 600 nm became 90% or less. In order to separate the consequences of cyclization on the heat-induced dehydration and coil-globule transition through (i) the elimination of the strongly hydrophilic hydroxy end groups and (ii) conformational restriction of the cyclic topology, *T*_c_ for the linear hydroxy-terminated, methoxy-terminated, and cyclized species of the following four Pluronic copolymers: L35, L64, 10R5, and 17R4 were investigated (Figure 3, Table 1). For the ABA-type Pluronic, a significant decrease in *T*_c_ was observed for the cyclic species. For example, the *T*_c_ of ***c*-L35** and ***c*-L64** at 64 and 56 °C, respectively, were comparably lower than their linear hydroxy-terminated counterparts (***l*-L35(OH)**, 82 °C; ***l*-L64(OH)**, 64 °C). In contrast, cyclization of the BAB-type copolymers resulted in comparable *T*_c_ to their corresponding linear hydroxy-terminated species. For instance, the *T*_c_ of ***l*-10R5(OH)** and ***c*-10R5** were 72 and 70 °C, respectively, while that of ***l*-17R4(OH)** and ***c*-17R4** were 49 and 53 °C, respectively. Interestingly, the linear methoxy-terminated species (***l*-L35(OMe)**, 81 °C; ***l*-L64(OMe)**, 68 °C; ***l*-10R5(OMe)**, 66 °C; ***l*-17R4(OMe)**, 44 °C, Table 1) gave completely different *T*_c_ values and tendencies to those of cyclized species, where comparable or increased *T*_c_ were obtained for the ABA-type copolymers, in contrast to significant decreases in *T*_c_ for the BAB-type copolymer solutions. The obtained results suggest a clear distinction in the topology effect of cyclization to that of chemical modification of the chain ends on the clouding phenomena.

To rationally explain the differences in transmittance changes upon temperature elevation of the ABA-type Pluronic copolymer solutions, an interpretation of the clouding mechanism is required. First, both linear hydroxy- and methoxy-terminated species of L35 and L64 (***l*-L35(OH)**, ***l*-L35(OMe)**, ***l*-L64(OH),** and ***l*-L64(OMe)**) displayed similar %T profiles, where the transmittance drastically drops at the *T*_c_. Clouding behavior of Pluronic copolymers to arise from phase transition induced through the dehydration and conformational change of the PEG segment is widely known [24,31], and our results suggest methylation of the chain-ends does not affect this mechanism. In contrast, the %T profiles of the cyclized species of the ABA-type copolymer solutions indicate a distinct aggregation upon temperature elevation; the transition of the cyclized species took place over a wider temperature range compared to their linear counterparts (Figure 3a,b), especially for ***c*-L64**. A similar phenomenon was reported on the phase transition of cyclic poly(*N*-isopropylacrylamide) (PNIPAM), explained to be caused by the disturbed packing of the polymer chains upon a coil-to-globule transformation due to the lack of chain ends [57,58], suggesting an analogous behavior to be exhibited in our system.

BAB-type copolymers are rationally expected to form flower-like micelles in water, where a fraction of the copolymer chains are expected to exist as or become “dangling chains” upon temperature elevation. The presence of “dangling chains” in these flower-like micelles is explained to act as inter-micellar bridging agents, which cause macroscopic aggregation, where the cyclization results in the elimination of this agglomeration mechanism [22,59]. In the case of the present Pluronic copolymers, the contribution from inhibition of the inter-micellar bridging was possibly observed for ***c*-17R4**, resulting in a slight increase in its *T*_c_. On the other hand, the decreased *T*_c_ value for the linear methoxy-terminated species can be interpreted as a consequence of reduced solvation of the hydrophobic segment of the polymer, resulting in enhanced micellar bridging agglomeration to precede the phase transition.

**Figure 4:**
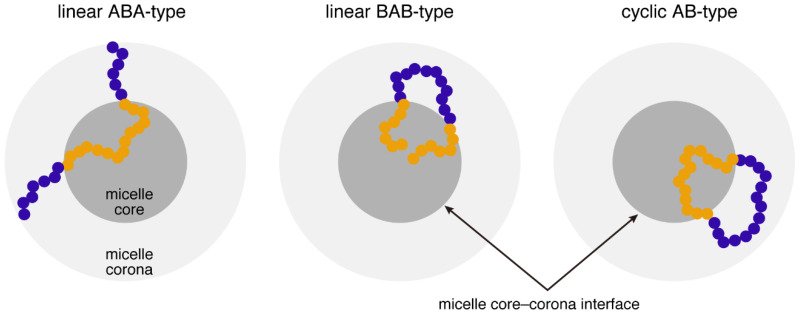
Schematic illustration of the expected conformations of linear ABA-type (**left**), linear BAB-type (**center**), and cyclized (**right**) copolymers in the micellar state. Cyclization results in the fixture of the PEG segment ends at the core–corona interface, and thus, is expected to affect the phase transition phenomena more prominently for the ABA-type copolymer.

When the chain conformations and the freedom of each block in the micellar state are taken into account, linear triblock ABA-, BAB-type, and cyclic AB-type species are all expected to exhibit different characteristics (Figure 4). For example, the two PEG segments of linear ABA-type copolymers are only attached to the core–corona interface at one end of each block, in contrast to the looped PEG corona of linear BAB-type and cyclized species, with both PEG block-ends attached to the core. This is expected to produce significant differences in the conformational freedom of the PEG blocks, which may influence the hydration and *T*_c_. Thus, the significant decrease in *T*_c_ upon cyclization observed only for the ABA-type copolymers can be explained as the result of the fixture of the free block ends at the core–corona interface, restricting their conformation.

To test this hypothesis of the restricted chain conformation of PEG being a factor behind the reduction in *T*_c_, temperature-dependent transmittance measurements were carried out for the linear hydroxy-terminated and cyclized species of PEG homopolymers (***l*-PEG(OH)** and ***c*-PEG**, respectively) (Table 1, Figure 3e). PEG solution samples were prepared at polymer concentrations of 10 g/L in a NaH_2_PO_4_ aqueous solution (NaH_2_PO_4_ concentration of 250 g/L) to induce phase transition under the boiling point of water [38,39]. As hypothesized, the cyclized species displayed lowered *T*_c_ compared to its linear counterparts (***l*-PEG(OH)**, 65 °C; ***c*-PEG**, 59 °C), indicating conformational restriction via cyclization to influence its solvation. This result bears a resemblance to thermal phase transition studies carried out for linear and cyclic species of PNIPAM [57,58,60]. Although the *T*_c_ of the cyclic PNIPAM samples was found to have concentration dependency and, therefore, not always be lower than that of their linear counterparts, reduced enthalpy changes during the clouding process were prevalent. These results, indicative of weakened hydrogen bonding interactions between the polymer and solvent water molecules for the cyclized species, have been explained as a consequence of either restrictions on the backbone conformation and/or steric constraints. A similar case could be assumed for the cyclized species of PEG and Pluronic copolymers investigated in this study.

### 3.4. Dynamic Light Scattering (DLS)

**Figure 5:**
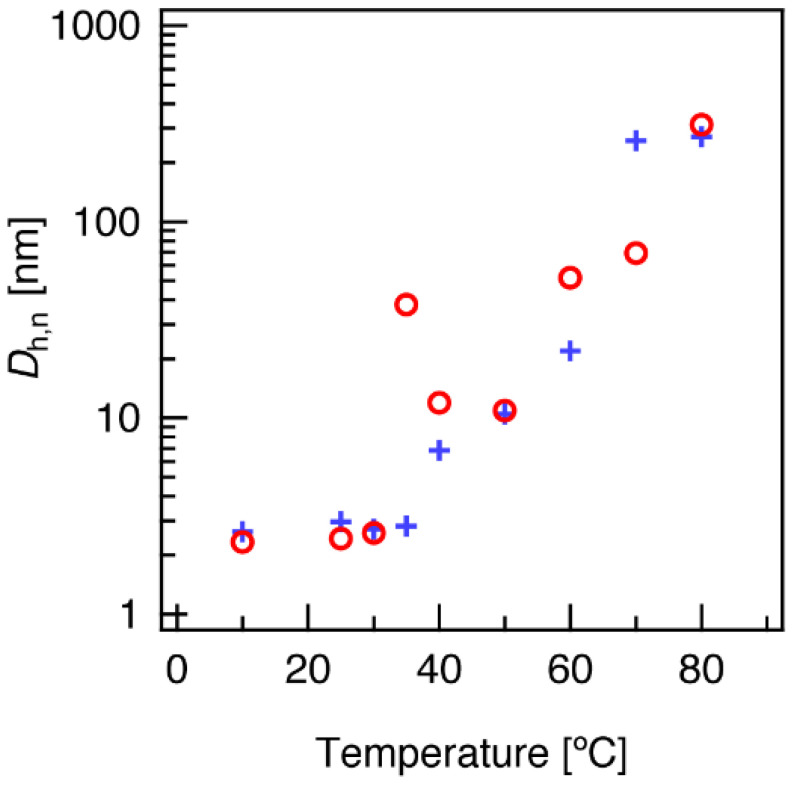
Number-average hydrodynamic diameter (*D*_h,n_) obtained from dynamic light scattering (DLS) at various temperatures for 10 g/L aqueous solutions of ***l*-L64(OH)** (blue cross) and ***c*-L64** (red circle).

In the temperature-dependent %T profiles of ***c*-L64** (Figure 3b), a plateau of around 30 %T was reached after the initial gradual transmission drop, from approximately 60 to 80 °C. A second sharp drop in %T was found over 80 °C, after which the solution became a 0 %T value. This implies a distinction in the thermally induced aggregation and/or phase separation phenomena of Pluronic L64, originating from its cyclic topology. In light of the anomalous association observed for ***c*-L64**, a structural investigation into the micellar aggregate size was conducted for L64 using DLS. The number-average hydrodynamic diameter (*D*_h,n_) at finite concentration for 10 g/L solutions of ***l*-L64(OH)** and ***c*-L64** at various temperatures is shown in Figure 5, while their number and intensity distribution profiles are shown in Figure S6. In consistency with the dye solubilization measurements, *D*_h,n_ of both ***l*-L64(OH)** and ***c*-L64** smaller than 5 nm indicate the copolymers to be mostly molecularly dissolved as the unimer state below *T*_CMT_, with some large aggregates as indicated from multiple peaks in the intensity distribution (Figure S6). Following temperature elevation, an evident size increase and the unification of multiple peaks in the intensity distribution were observed at 40 °C for ***l*-L64(OH)** and at 35 °C for ***c*-L64**. While these suggest micellization around the corresponding temperatures, the apparent size for ***c*-L64** at 35 °C indicates the cyclized species initially form larger aggregates around its *T*_CMT_, which break down into smaller micelles upon further heating. At 40 and 50 °C, both linear and cyclized copolymer systems displayed similar *D*_h,n_ of around 10–20 nm (*D*_h,n_ at 40 °C: ***l*-L64(OH)**, 7 nm; ***c*-L64**, 12 nm), indicating the formation of well-defined micellar aggregates.

Temperature elevation for ***l*-L64(OH)** revealed a gradual increase in micelle size from 40 to 60 °C, followed by a sudden increase in the mean value and distribution of *D*_h,n_ from 22 nm at 60 °C to 260 nm at 70 °C, which underwent no further significant change upon temperature elevation to 80 °C, where *D*_h,n_ of 280 nm, was observed. The gradual increase in *D*_h,n_ between 40 and 60 °C is in coincidence with previous studies, where aggregation number and dimensions of Pluronic micelles are known to slightly increase upon temperature elevation [24,44]. Furthermore, the sudden size increase from 60 °C to 70 °C is indicative of the clouding phenomena and is consistent with the temperature-dependent transmittance results, where a drop in light transmittance occurred for ***l*-L64(OH)** between 60 and 70 °C (Figure 3b).

In contrast to the results for ***l*-L64(OH)**, the ***c*-L64** system underwent a more complex change upon heating. Between 40 and 50 °C, its *D*_h,n_ remained constant (11–12 nm), and thus, the majority of the copolymer micelles were found to retain their particle size. On the other hand, the intensity distribution indicated the formation of larger aggregates around 50–100 nm at 50 °C (Figure S6d). Upon further temperature elevation to 60 °C, these aggregates became the major component of the system as seen in *D*_h,n_ (52 nm). However, at 60 °C, the presence of even larger aggregates (ca. 500 nm) was indicated in the intensity distribution. These aggregates are expected to be the cause of the first drop in light transmittance (Figure 3b). Moreover, the second transmittance drop at 80 °C likely arose from these species’ becoming the major component of the system. The above results suggest the cyclic topology influences the thermally induced aggregation of ***c*-L64**. Nevertheless, multi-step %T changes were not observed for the other Pluronic copolymer systems (***c*-L35**, ***c*-10R5,** and ***c*-17R4**), and thus, the effect of cyclization on the aggregation phenomena is also expected to be in relation to the block composition and sequence of the copolymer.

### 3.5. The Effect of Cyclization on the Critical Temperatures

**Figure 6:**
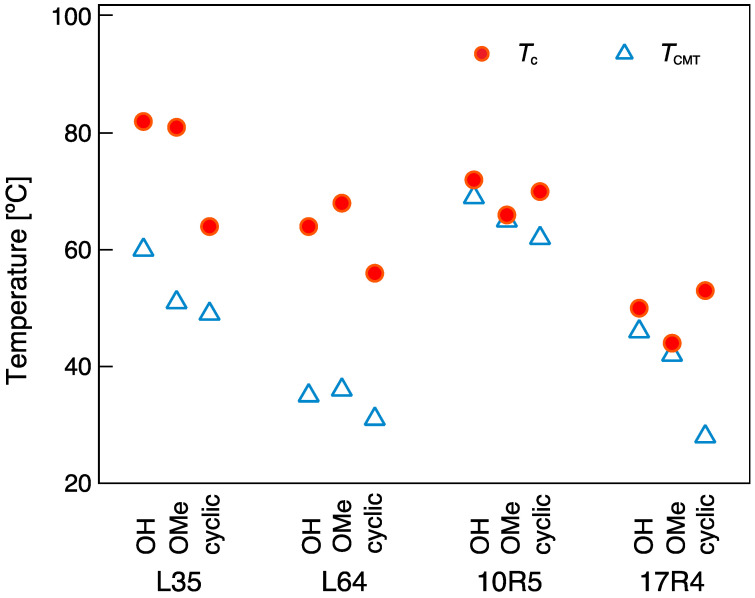
Critical micellization temperature (*T*_CMT_) and cloud point (*T*_c_) for 10 g/L solutions of Pluronic L35, L64, 10R5 and 17R4. Linear hydroxy-terminated species, linear methoxy-terminated and cyclized species are represented as **OH**, **OMe**, and **cyclic**, respectively.

From dye solubilization, transmittance, and DLS measurements, cyclization was found to both affect the micellization and clouding behavior of Pluronic, each to a different degree for the four copolymer species explored in this study. A comparison of the critical temperatures for the linear hydroxy-terminated, linear methoxy-terminated, and cyclized species of the various Pluronic copolymers is shown in Figure 6. The micellar region, i.e., the temperature region above *T*_CMT_ and below *T*_c_, differs depending on the block sequence of the Pluronic copolymers. The micellar region of the ABA-type Pluronic L35 and L64 is relatively large, and while both methylation of the chain-ends and cyclization result in a general downward shift of *T*_CMT_, *T*_c_ was significantly affected only by cyclization. For the linear BAB-type copolymers, *T*_CMT_ and *T*_c_ of the linear hydroxy-terminated and methoxy-terminated species are almost overlapping; thus, their micellar region is extremely small, or rather, they form a randomly cross-linked micellar network at this concentration [61]. The cyclized species, however, displayed prominently decreased *T*_CMT_ and increased *T*_c_ compared to the linear methoxy-terminated species, resulting in the expansion of the micellar region. Summarizing the above, the topology and the chain-ends considerably affect their critical temperatures in relation to the block sequence of the linear species.

## 4. Conclusions

The effects of cyclization were observed for Pluronic copolymers in their bulk solution properties. Investigation into the thermodynamic properties of micellization revealed a downward shift in *T*_CMT_ with decreased Δ*H*_mic_ and Δ*S*_mic_ for the cyclized species. This is believed to be due to a more effective shielding of the hydrophobic PPG segments by the hydrophilic PEG segments in the cyclized species in the unimer state. Moreover, %T and DLS measurements revealed a contrasting effect on the clouding phenomena of the cyclized Pluronic micelles depending on their block sequence, where a pronounced decrease in *T*_c_ was observed for the ABA-type copolymers. A comparison with the methoxy-terminated linear species indicated the effect of cyclization to differ from the contribution from the elimination of the hydrophilic chain-end groups. Thus, the interpretation of the effect of cyclization on its phase transition behavior was discussed as arising from conformational restrictions and/or steric constraints of the polymer chains induced from the cyclic topology. A comprehensive understanding of the effects of cyclization allows the utilization of polymer topology to be a viable option in the rational design of polymeric materials and thus contributes to the fabrication of novel functional materials and their applications.

## Figures and Tables

**Table 1:** Properties of Hydroxy- and Methoxy-Terminated Linear and Cyclized PEG and Pluronic Copolymers and Their Solutions’ Thermodynamic Parameters.

*Topology*-Pluronic (Chain-End)	Composition	*M*_n_ (g/mol)	*T*_CMT_ at 10 g/L (°C)	Δ*H*_mic_ (kJ/mol)*^a^*	Δ*G*_mic_ (kJ/mol) *^b^*	Δ*S*_mic_ (J/(mol K)) *^b^*	*T*_c_ at 10 g/L (°C)
***l*-L35(OH)**	(EG)_11_–(PG)_16_–(EG)_11_	1900	58	91 ± 4	–26.1	352	82
***l*-L35(OMe)**			51	96 ± 5	–25.4	374	81
***c*-L35**			45	77 ± 3	–25.2	319	64
***l*-L64(OH)**	(EG)_13_–(PG)_30_–(EG)_13_	2900	35	182 ± 10	–24.6	670	64
***l*-L64(OMe)**			36	183 ± 9	–24.7	672	68
***c*-L64**			31	156 ± 2	–24.3	593	56
***l*-10R5(OH)**	(PG)_8_–(EG)_22_–(PG)_8_	2000	69	145 ± 6	–26.9	505	72
***l*-10R5(OMe)**			65	152 ± 20	–26.5	530	66
***c*-10R5**			62	94 ± 7	–25.9	365	70
***l*-17R4(OH)**	(PG)_14_–(EG)_24_–(PG)_14_	2700	46	173 ± 3	–26.4	625	49
***l*-17R4(OMe)**			42	230 ± 30	–26.1	804	44
***c*-17R4**			28	77 ± 5	–24.9	339	53
***l*-PEG(OH)**	(EG)_45_	2000	-	-	-	-	65
***c*-PEG**			-	-	-	-	59

*^a^* Average molecular weights reported from the manufacturer were used for the calculation of thermodynamic parameters of micellization. *^b^* Thermodynamic parameters obtained for 10 g/L solutions at *T*_CMT_.

## Data Availability

The data presented in this study are available on request from the corresponding author.

## Supplementary Materials

The following supporting information can be downloaded at: https://www.mdpi.com/article/10.3390/polym14091823/s1, Figure S1: SEC traces of Pluronic and PEG samples, Figure S2: ^1^H NMR spectra of Pluronic and PEG samples, Figure S3: ^13^C NMR spectra of Pluronic and PEG samples, Figure S4: Temperature-dependent transmittance of ***l*-L64(OH)**, Figure S5: Temperature-dependent absorption intensity of DPH in the presence of Pluronic samples, Figure S6: Number and intensity distribution obtained from DLS.
