# Supplementary material for: Topology and Sequence-Dependent Micellization and Phase Separation of Pluronic L35, L64, 10R5, and 17R4: Effects of Cyclization and the Chain Ends
Source: Polymers (Basel). 2022 Apr 29;14(9):1823. doi: 10.3390/polym14091823 (PMC9105568; doi:10.3390/polym14091823)
Supplement: Supplementary file 1 [file polymers-14-01823-s001.zip › polymers-1692867-supplementary.pdf]

Supplementary Materials

Topology- and Sequence-Dependent Micellization and Phase Separation of Pluronic L35, L64, 10R5, and 17R4: Effects of Cyclization and the Chain Ends

Tomohisa Watanabe [1], Yubo Wang [1], Tomoko Ono [2], Satoru Chimura [1], Takuya Isono [2], Kenji Tajima [2], Toshifumi Satoh [2], Shin-ichiro Sato [2], Daichi Ida [3] and Takuya Yamamoto [2,*]

[1] Graduate School of Chemical Sciences and Engineering, Hokkaido University, Sapporo, Hokkaido 060-8628, Japan; tomohisa_watanabe@eis.hokudai.ac.jp (T.W.); ougyokuhaku@eis.hokudai.ac.jp (Y.W.); a1fi_7ht1@outlook.jp (S.C.)

[2] Division of Applied Chemistry, Faculty of Engineering, Hokkaido University, Sapporo, Hokkaido 060-8628, Japan; t-ono1808@eng.hokudai.ac.jp (T.O.); isono.t@eng.hokudai.ac.jp (T.I.); ktajima@eng.hokudai.ac.jp (K.T.); satoh@eng.hokudai.ac.jp (T.S.); s-sato@eng.hokudai.ac.jp (S.S.)

[3] Department of Polymer Chemistry, Graduate School of Engineering, Kyoto University, Katsura, Kyoto 615-8510, Japan; ida@molsci.polym.kyoto-u.ac.jp

* Correspondence: yamamoto.t@eng.hokudai.ac.jp

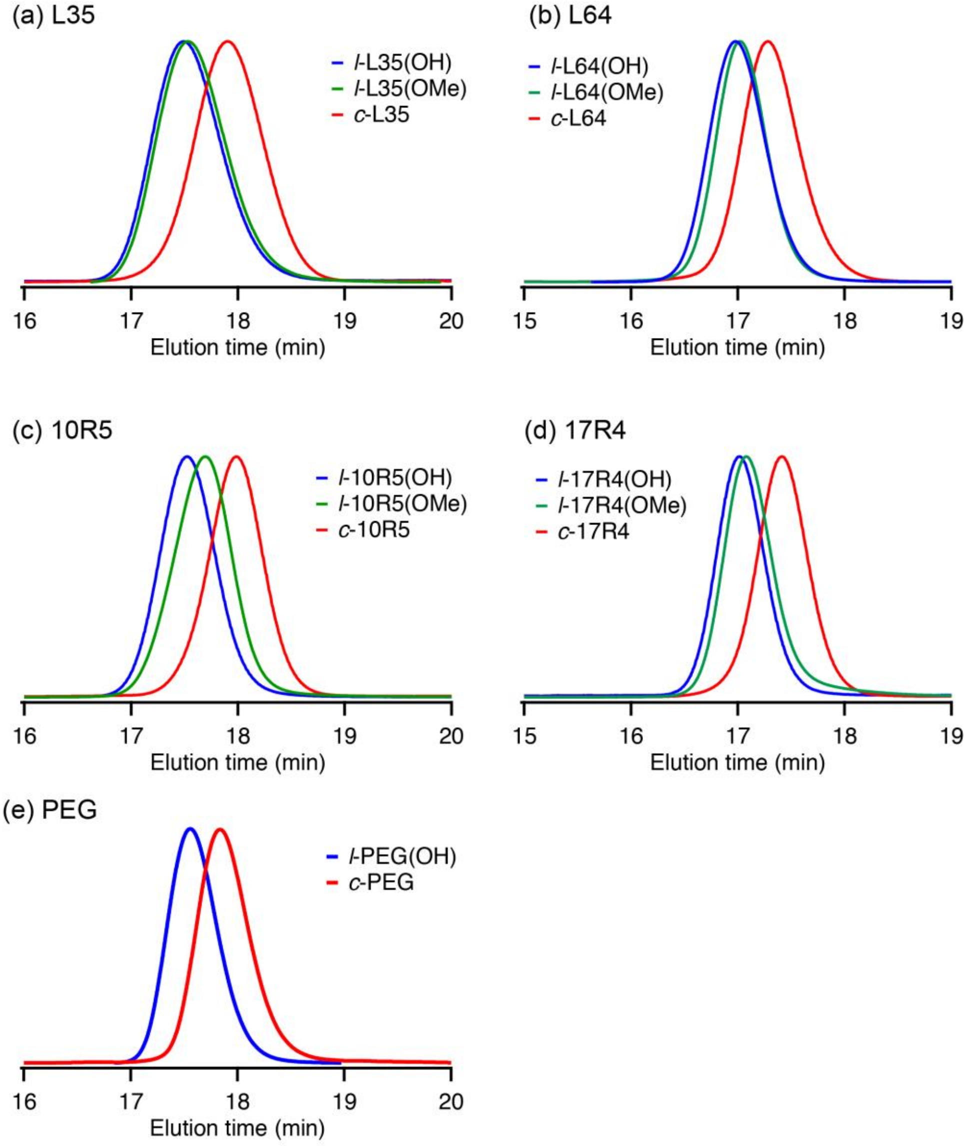

Figure S1. (a) SEC traces of linear hydroxy-terminated (blue), methoxy-terminated (green), and cyclized (red) L35, indicated as ***l*-L35(OH)**, ***l*-L35(OMe)** and ***c*-L35**, respectively. Those of (b) L64, (c) 10R5, (d) 17R4 and (e) PEG are also shown. Longer elution times were observed for the cyclized species due to reduction in the hydrodynamic volume.

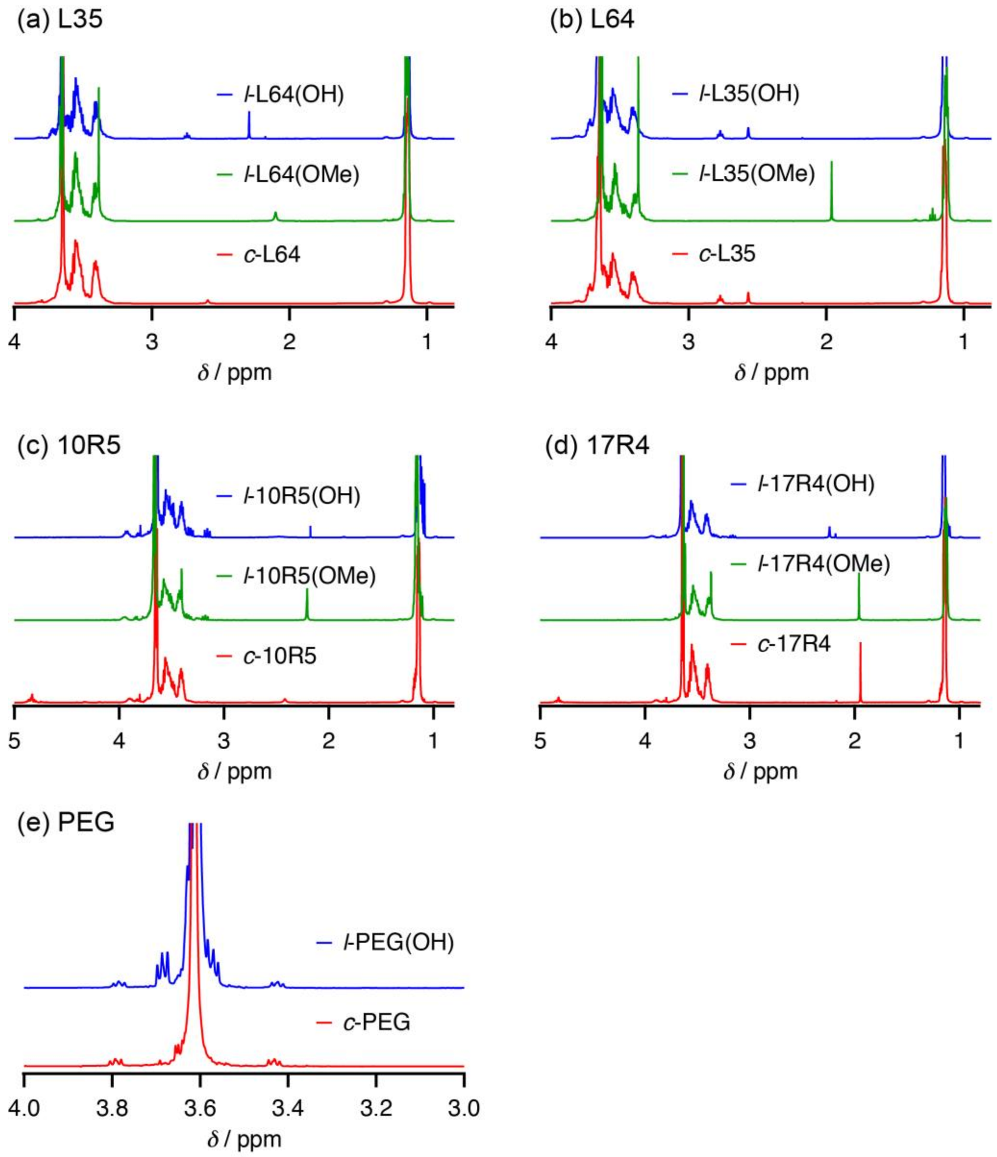

Figure S2. (a) ^{1}H NMR spectra of linear hydroxy-terminated (blue), methoxy-terminated (green), and cyclized (red) L35, indicated as ***l*-L35(OH)**, ***l*-L35(OMe)** and ***c*-L35**, respectively. Those of (b) L64, (c) 10R5, (d) 17R4 and (e) PEG are also shown.

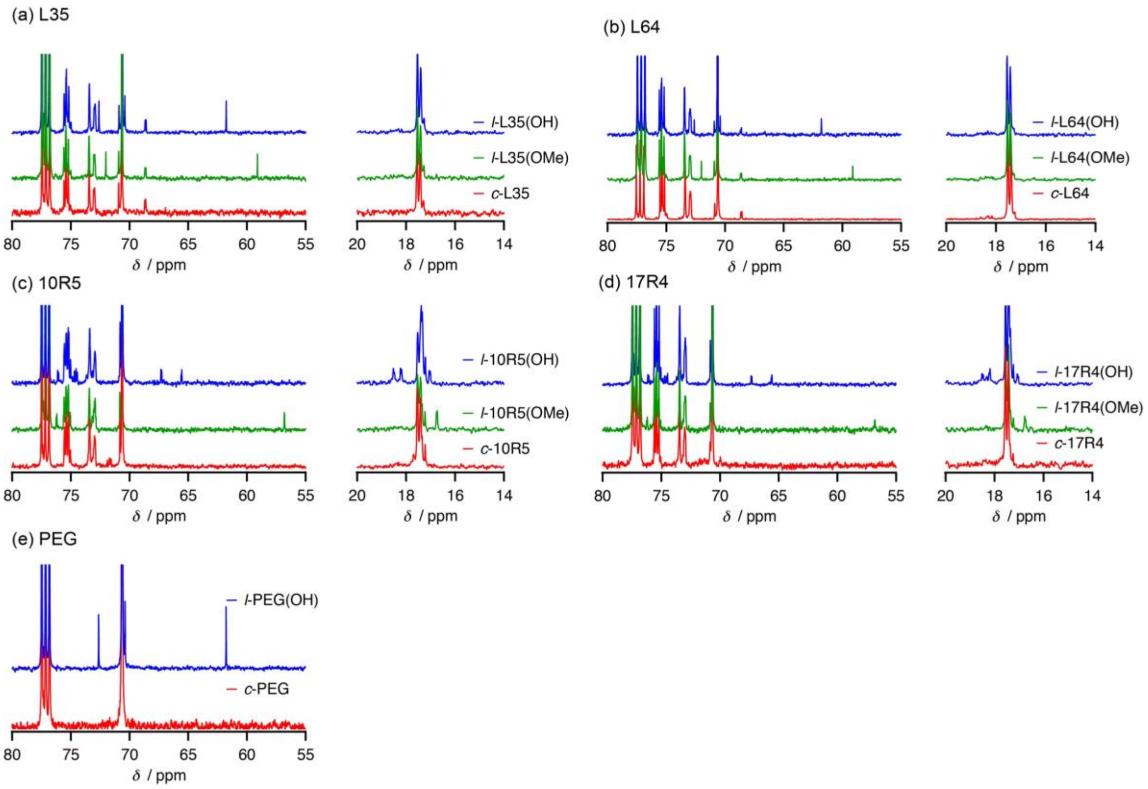

Figure S3. (a) ^{13}C NMR spectra of linear hydroxy-terminated (blue), methoxy-terminated (green), and cyclized (red) L35, indicated as ***l*-L35(OH)**, ***l*-L35(OMe)** and ***c*-L35**, respectively. Those of (b) L64, (c) 10R5, (d) 17R4 and (e) PEG are also shown.

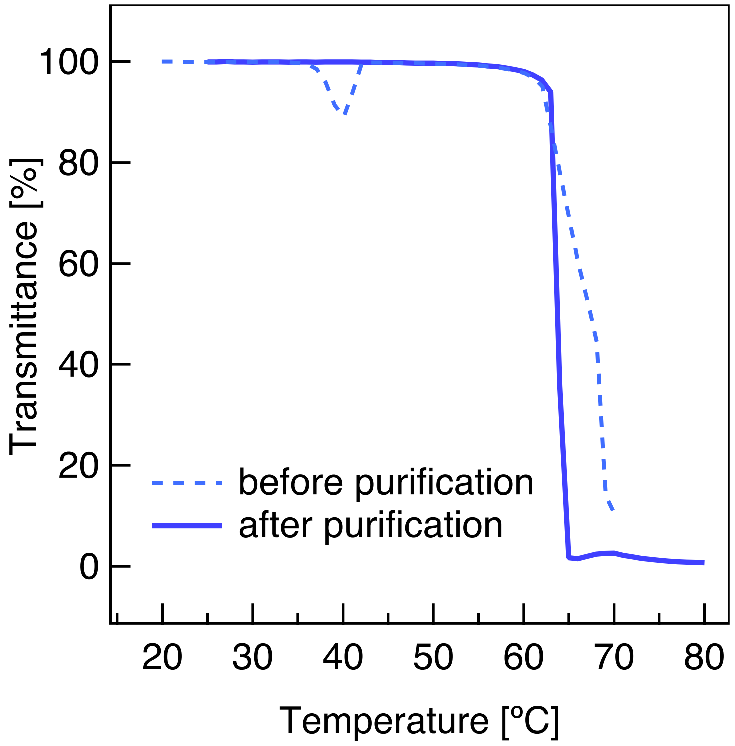

Figure S4. Temperature-dependent %T for 10 g/L aqueous solutions of ***l*-L64(OH)** using copolymers before (dotted line) and after (solid line) purification through *n*-hexane washing

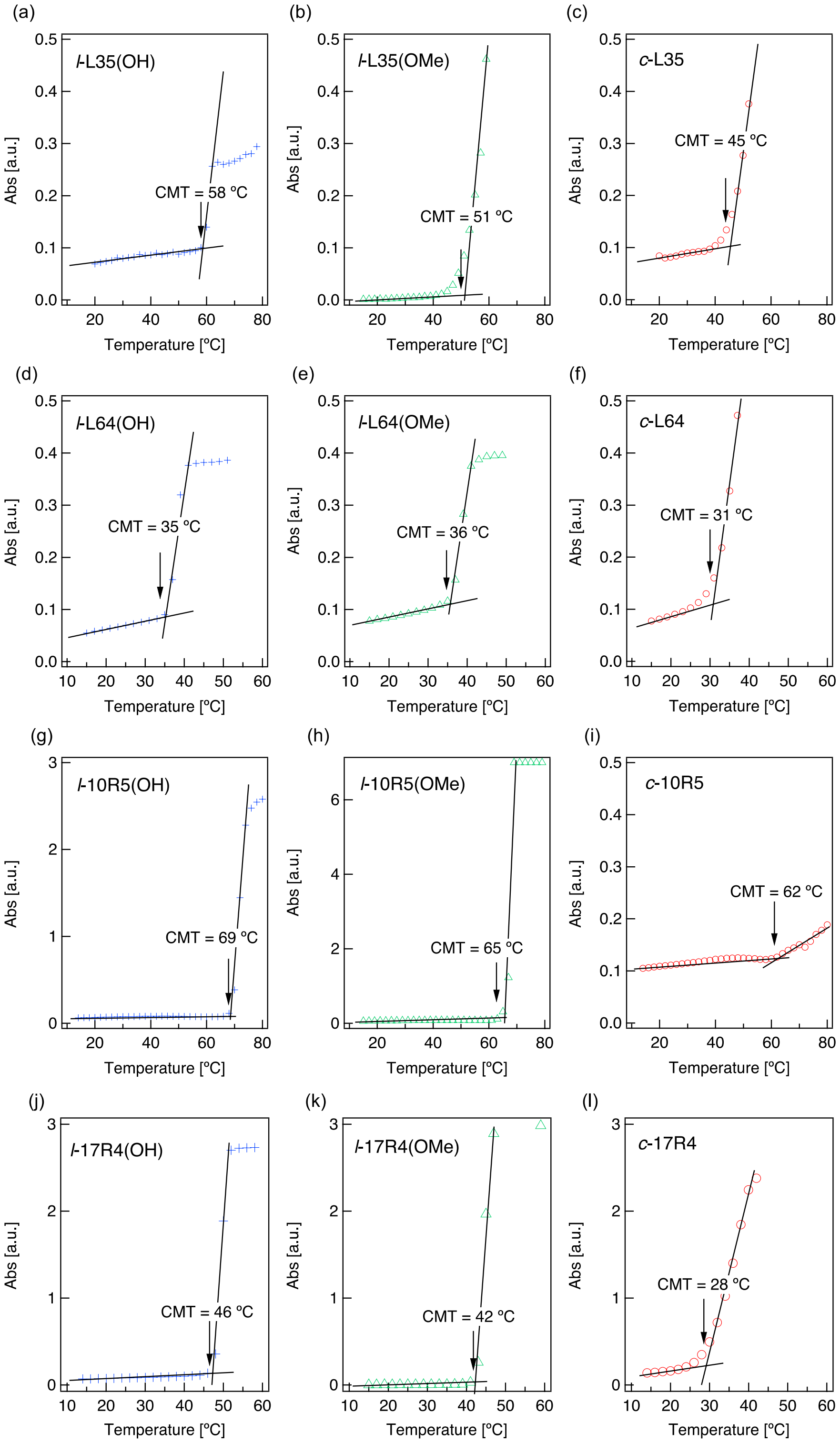

Figure S5. Temperature-dependent absorption intensity of DPH ($\lambda_{\mathrm{max,\,DPH}}$ = 356 nm) for 10 g/L solutions of (a) ***l*-L35(OH)**, (b) ***l*-L35(OMe)** and (c) ***c*-L35**. Those of L64, 10R5, and 17R4 are also shown in (d)–(l). Critical micellization temperature (T_{CMT}) was determined as the intersection temperature where an increase in the slope of the absorption intensity was observed.

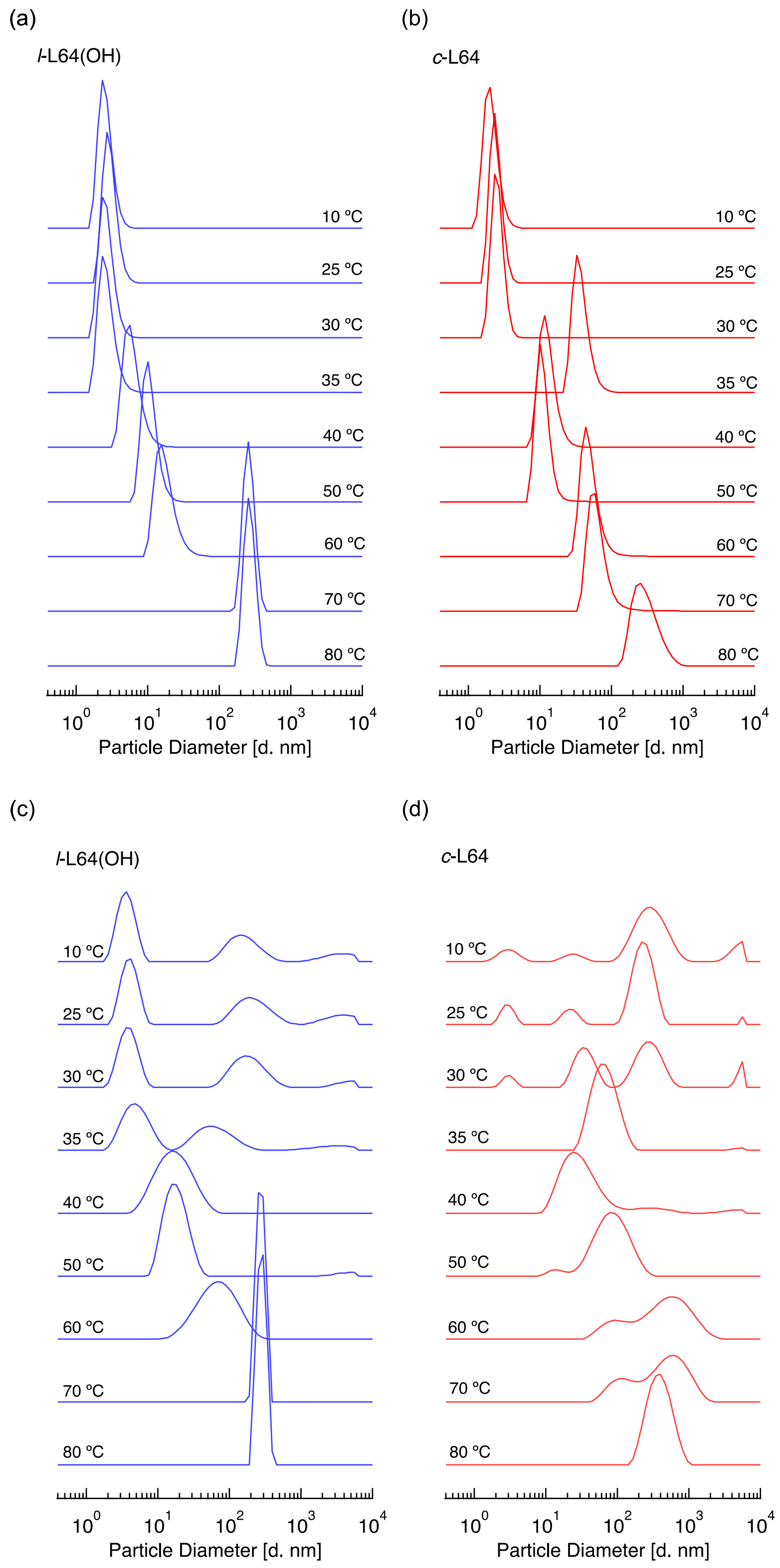

Figure S6. Number distribution profiles for 10 g/L aqueous solutions of (a) ***l*-L64(OH)** and (b) ***c*-L64** obtained from dynamic light scattering (DLS) at various temperatures. Corresponding intensity distribution profiles are shown in (c) and (d), respectively.
